# Dengue Deaths: Associated Factors and Length of Hospital Stay

**DOI:** 10.1155/2016/6807674

**Published:** 2016-07-04

**Authors:** S. Pooransingh, S. Teelucksingh, I. Dialsingh

**Affiliations:** ^1^Faculty of Medical Sciences, The University of the West Indies, St. Augustine, Trinidad and Tobago; ^2^Department of Mathematics and Statistics, Faculty of Science and Technology, The University of the West Indies, St. Augustine, Trinidad and Tobago

## Abstract

*Background*. Dengue continues to pose a public health problem globally.* Objective*. To review factors associated with patients who died from dengue in Trinidad.* Methods*. A retrospective case note review of hospitalized patients who died during 2001 to 2010.* Results*. A total of 23 cases were identified: 13 males, 10 females—12 East Indians, 9 Africans, and 2 unknown. More than half (*n* = 17) were over 40 years of age with 10 being over 60 years of age; three were children. A falling platelet count was observed in 16 while 18 patients had a low normal haematocrit. There was a significant association of ethnicity, hypertension, and diabetes with length of hospital stay.* Conclusions*. The study sample included 10 patients over 60 years of age. Patients with diabetes and hypertension and patients of East Indian origin appeared to have a shorter hospital stay prior to death.

## 1. Introduction

Dengue has been reported in the literature since the 18th century [1, 2] and continues to present significant morbidity and mortality upon populations globally. It is estimated that 50 million infections occur annually in about 100 countries [2]. The* Aedes aegypti* mosquito, the main vector of the four strains of dengue virus (DENV1 to DENV4), is widely distributed across tropical and subtropical zones. The mosquito has spread globally aided by increased trade and travel [2]. Severe dengue, recognised in the 1950s in the Philippines and Thailand [3], was first reported in the Western Hemisphere in Cuba in 1981 [4] but now affects most countries in Central and South America where it is a leading cause of hospitalization and death. The majority of infections are asymptomatic, but all four viruses are associated with dengue fever and a minority of these progress to dengue haemorrhagic fever (DHF) and dengue shock syndrome (DSS) [5].

Trinidad and Tobago is a twin island nation located within the Caribbean chain of islands, with a population of approximately 1.3 million persons. The first isolate of a dengue virus DENV2 in the Americas occurred in Trinidad in 1953 [5, 6] and the first cases of dengue haemorrhagic fever (DHF) and dengue shock syndrome (DSS) were reported among adults in Trinidad in 1992/1993 [7]; paediatric cases were later reported [8]. Dengue presented as outbreaks of DENV2 for a number of years until urbanization and population growth and travel facilitated the occurrence of epidemic dengue in areas outside of South East Asia. In the Americas, between 1947 and 1963, there was no evidence of epidemic dengue; in 1963 and 1977 there were epidemics of DENV3 and DENV1, respectively. The first major DHF/DSS outbreak occurred in 1981 [5].

It has been reported that comorbidities in patients with dengue result in complications leading to death [9]. A recent literature review on noncommunicable disease comorbidities and dengue revealed that comorbidities, that is, cardiovascular disease, stroke, diabetes, renal disease, respiratory disease, and old age, may contribute to severe dengue [10].

The purpose of this study was to simply review the characteristics of the patients who died from dengue in Trinidad to determine if there were any identifiable factors that were associated with death from dengue.

## 2. Methods

A descriptive study was undertaken via a retrospective review of case notes from patients admitted to the public hospitals. Ethical approval was obtained from the University of the West Indies and the four regional health authorities in Trinidad where this work was done. The four major public hospitals in Trinidad were included. Patients who died from dengue between 2001 and 2010 inclusive were included. Patients were identified through death registers at each hospital by looking at the diagnosis fields for an entry labelled as dengue, DHF, or DSS. In Trinidad the case definitions utilised by physicians were in accordance with the 1997 WHO case classifications which distinguished dengue fever, dengue haemorrhagic fever, and dengue shock syndrome. These were reinforced by national guidelines developed by one of the authors and disseminated through the Ministry of Health. In 2009 when WHO changed their classification of dengue the Ministry of Health issued the new case definitions and hospitals displayed summaries of the guidelines on their clinic walls outlining the new case definitions and their management. Private General Practitioners and private hospitals were not included in this study since General Practitioners would usually refer their patients to public hospital; in addition, it was assumed that patients (including children) who present to private facilities would end up at the public hospitals since the facilities at many private hospitals are inadequate to care for very ill patients and furthermore they would be costly to the patient since it is often the case that patients who initially present to private hospitals transfer to the public system when the daily cost becomes unaffordable over time.

The number of deaths obtained through the methodology adopted in this study was validated by comparing with PAHO [11] data for Trinidad for the same time period rather than accessing the Central Statistical Office as the Central Statistical Office transmits their data to the Ministry of Health who then sends the data onwards to PAHO.

The patient registration numbers were obtained from the death register and the medical records staff at each hospital retrieved the case notes using the patient registration numbers. Medical case notes were reviewed by one of the authors who visited each hospital and extracted data using a data collection form. Statistical analyses were performed to determine if there were any associations between the time from admission to death (length of stay) and factors such as ethnicity, age, gender, haematological parameters, and presence of comorbidities, specifically diabetes and hypertension. We acknowledge that length of stay as an outcome variable is an indicator of patient care and treatment; however we assume that all patients with a diagnosis of dengue are treated according to the WHO 2009 clinical guidelines and hence care would be administered according to need. This approach was validated by Toledo et al. who utilised this outcome variable along with mortality in their recent systematic review on comorbidities and severe dengue [10].

## 3. Results

The study retrieved 23 case notes for patients who died from dengue during the study period. Males (*n* = 13) outnumbered females (*n* = 10) and East Indian Trinidadians (*n* = 12) outnumbered African Trinidadians (*n* = 9); ethnicity data were missing in 2 cases. Three cases were children (ages 1/12, 5/12, and 12 years). The range for adults was 21–88 years (mean 55.9, SD 21.7). More than 70% of cases were over 40 years of age. Ten patients had a history of diabetes and six, hypertension with five patients recorded as having both diabetes and hypertension. Thrombocytopenia was observed in 16 patients. Haemoconcentration was recorded in one case. Eighteen cases had a low or normal haematocrit on admission and during hospitalization (missing data *n* = 4).

Fisher's exact test revealed a significant association between ethnicity and time from admission to death (*p* value = 0.0014). There were significant differences in the proportion of inpatients with diabetes (*p* value = 0.0237), hypertension (*p* value = 0.0018), and the presence of both (*p* value = 0.021) and length of stay. There was no association between platelet levels nor age and length of stay.  Table 1 shows the findings.

## 4. Discussion

In the 1950s and 1960s dengue was mostly described in children in South East Asia. However spread into other regions including the Caribbean islands was facilitated by human migration, urban development, and the creation of artificial reservoirs such as used tyres [10]. Dengue is therefore now hyperendemic in the Caribbean islands including Trinidad with the circulation of all four serotypes and the potential for epidemics. Dengue is also increasingly seen in older adults who are also the population subgroup experiencing noncommunicable diseases such as diabetes, hypertension, and cancers. It is hypothesised that in older adults comorbidities predispose to more severe forms of dengue [10].

Over the 10-year period reviewed, 23 deaths from dengue were recorded in public hospitals. Comparison with PAHO data revealed 18 deaths for Trinidad and Tobago in the same time period [11] compared with our 23 cases. This was on a background of 16297 clinically diagnosed dengue cases (362 laboratory confirmed). Five hundred cases of dengue haemorrhagic fever and dengue shock syndrome (and since 2010, severe dengue) were observed over the ten-year period with 18 deaths. Over the 10-year period the numbers of DHF/DSS were seen to decrease from 86 in 2001 to 3 in 2010. Many initiatives aimed at dengue prevention and control are ongoing in Trinidad as part of the Integrated Management Strategy for Dengue, including research on the vector [12].

Our study revealed more patients of East Indian compared with African origin, a pattern previously reported from Trinidad [7]. The majority of our patients were over 40 years with 10 patients being over 60 years of age. This contrasts data from South East Asia where death from dengue is particularly high among children [13]. Toledo et al. recently described the occurrence of severe dengue in older adults with comorbidities which gives support to our study findings [10].

A Singaporean study showed Chinese ethnicity, female gender and age group 30–49 years, diabetes, or diabetes and hypertension to be associated with greater risk of DHF in a serotype 2 outbreak [14]; our study showed an association with ethnicity, diabetes, and hypertension, but not with gender. A previous study finding in Trinidad of more severe disease in East Indian patients may explain the shorter length of hospital stay, although time from symptom onset to admission may also play a role; however this parameter was incomplete in more than 50% of records which prevented further analysis. Figueiredo et al. [15] also found an association between reported diabetes and DHF and Saqib et al. also found an association with hypertension and dengue deaths [16].

Lum et al. [17] found that the strongest indicator for haemorrhage was prolonged duration of shock and a haematocrit in the low to normal range during the period of shock, thereby recommending early recognition of shock and correction of circulatory status. There was no record of overt bleeding in the case notes in our study, so it is possible that occult bleeding may have been present in some of our cases as 78.2% had a low/normal haematocrit during hospitalization.

Background rates of diabetes and hypertension are high in the Trinidadian population [18] and dengue deaths among those with these comorbidities may be coincidental; it is not possible to conclude in this study whether these comorbidities confer added risk for complicated dengue or death; however the findings have been mirrored in larger studies [9, 13, 14], and more recently the systematic review by Toledo et al. supports the occurrence of severe dengue in older adults with comorbidities seen in our study [10].

## 5. Limitations

The retrospective nature of the study and thus incompleteness of medical records led to missing data for some parameters. Our sample size was small at *n* = 23; however data from PAHO indicate a similar picture in terms of numbers. The population of the island is small estimated at 1.3 million persons.

## 6. Conclusion

This is the first study on dengue deaths in our setting. We found significant associations between East Indian ethnicity and the presence of diabetes and hypertension and length of hospital stay in our study sample. A prospective multicentre study, since our numbers are small, to confirm these findings and to determine the role of comorbidities and their potential complications in dengue morbidity and mortality would be useful in our setting where prevalence rates of diabetes and hypertension are high. Such information would be useful to public health practitioners in designing preventive health educational initiatives and to clinicians in their case management.

## Figures and Tables

**Table 1:** Admission time to death (days) versus risk factors.

Risk factor		Admission time to death (days)			*p* values
		0–3 days	4–9 days	Over 9 days	
Gender	Female	4 (40.0%)	3 (30.0%)	3 (30.0%)	0.8636
	Male	5 (41.7%)	5 (41.7%)	2 (16.7%)	

Ethnicity	African	0 (0.0%)	5 (62.5%)	3 (37.5%)	0.0014^*∗*^
	East Indian	9 (75.0%)	1 (8.3%)	2 (16.7%)	

Age (years)	0–20	3 (100.0%)	0 (0.0%)	0 (0.0%)	0.3986
	21–50	2 (33.3%)	3 (50.0%)	1 (16.7%)	
	Over 50	4 (30.8%)	5 (38.5%)	4 (30.8%)	

Presence of diabetes	Yes	2 (20.0%)	3 (30.0%)	5 (50.0%)	0.0237^*∗*^
	No	7 (77.8%)	2 (22.2%)	0 (0.0%)	

Presence of hypertension	Yes	0 (0.0%)	3 (50.0%)	3 (50.0%)	0.0018^*∗*^
	No	9 (81.8%)	2 (18.2%)	0 (0.0%)	

Presence of both diabetes and hypertension	Yes	0 (0%)	2 (40%)	3 (60%)	0.021^*∗*^
	No	9 (52.9%)	6 (35.3%)	2 (11.8%)	

Platelet level	Decreased	7 (43.8%)	6 (37.5%)	3 (18.8%)	0.6698
	Normal	2 (40.0%)	1 (20.0%)	2 (40.0%)	

*p* values for Fisher's exact test to test whether there is an association between each variable and admission time to death. ^*∗*^Significant at the 0.05 level.
